# How voltage-gated calcium channels gate forms of homeostatic synaptic plasticity

**DOI:** 10.3389/fncel.2014.00040

**Published:** 2014-02-14

**Authors:** C. Andrew Frank

**Affiliations:** Department of Anatomy and Cell Biology, University of Iowa Carver College of MedicineIowa City, IA, USA

**Keywords:** homeostatic synaptic plasticity, VGCCs, Ca_V_1 channels, Ca_V_2 channels, neurotransmitter release, synaptic scaling, synaptic growth, calcium channelopathies

## Abstract

Throughout life, animals face a variety of challenges such as developmental growth, the presence of toxins, or changes in temperature. Neuronal circuits and synapses respond to challenges by executing an array of neuroplasticity paradigms. Some paradigms allow neurons to up- or downregulate activity outputs, while countervailing ones ensure that outputs remain within appropriate physiological ranges. A growing body of evidence suggests that homeostatic synaptic plasticity (HSP) is critical in the latter case. Voltage-gated calcium channels gate forms of HSP. Presynaptically, the aggregate data show that when synapse activity is weakened, homeostatic signaling systems can act to correct impairments, in part by increasing calcium influx through presynaptic Ca_V_2-type channels. Increased calcium influx is often accompanied by parallel increases in the size of active zones and the size of the readily releasable pool of presynaptic vesicles. These changes coincide with homeostatic enhancements of neurotransmitter release. Postsynaptically, there is a great deal of evidence that reduced network activity and loss of calcium influx through Ca_V_1-type calcium channels also results in adaptive homeostatic signaling. Some adaptations drive presynaptic enhancements of vesicle pool size and turnover rate via retrograde signaling, as well as *de novo* insertion of postsynaptic neurotransmitter receptors. Enhanced calcium influx through Ca_V_1 after network activation or single cell stimulation can elicit the opposite response—homeostatic depression via removal of excitatory receptors. There exist intriguing links between HSP and calcium channelopathies—such as forms of epilepsy, migraine, ataxia, and myasthenia. The episodic nature of some of these disorders suggests alternating periods of stable and unstable function. Uncovering information about how calcium channels are regulated in the context of HSP could be relevant toward understanding these and other disorders.

## Introduction

Many forms of neuroplasticity drive changes in synaptic outputs, and they are thought to underlie fundamental neurological phenomena, like learning. At the same time, stabilizing forms of neuroplasticity—collectively termed homeostatic synaptic plasticity (HSP)—work to ensure that neuronal outputs are maintained within physiologically appropriate levels. The study of HSP has shed considerable light on how neuronal stability is maintained. Perturbations of synaptic function can trigger homeostatic modulations in activity parameters such as presynaptic neurotransmitter release, neurotransmitter receptor expression, ion channel density, or conductance properties (Pérez-Otaño and Ehlers, 2005; Davis, 2006, 2013; Marder and Goaillard, 2006; Turrigiano, 2008; Maffei and Fontanini, 2009; Pozo and Goda, 2010; Watt and Desai, 2010; Marder, 2012; Turrigiano, 2012). The underlying molecular mechanisms that enable such change are critical (Lazarevic et al., 2013). How exactly do synapses detect challenges to their activity and then engage biological homeostats to correct errors? Some progress has been made in answering this question using a variety of models such as *Drosophila melanogaster* (Davis, 2013; Frank, 2014), crustaceans (Marder and Goaillard, 2006; Marder and Bucher, 2007; Marder, 2012), and rodents (Chen et al., 2014; Lee et al., 2014; Pribiag and Stellwagen, 2014; Thalhammer and Cingolani, 2014; Wenner, 2014; Whitt et al., 2014). In recent years, voltage-gated calcium channels (VGCCs or Ca_V_ channels) have emerged as critical for homeostatic control of synapse function in several experimental contexts.

When considering how Ca_V_ channels control any process (such as HSP), it is important to consider auxiliary interacting proteins, cellular context, and the high degree to which Ca_V_-driven processes are conserved across metazoans. It is known that neuronal Ca_V_ channels mediate cellular calcium entry and regulate activity-dependent processes such as neurotransmission, gene transcription, and intracellular signaling cascades (Catterall, 2000; Zamponi, 2005; Tedford and Zamponi, 2006; Catterall and Few, 2008; Dolphin, 2009; Currie, 2010; Lipscombe et al., 2013). Control of Ca_V_ function occurs at many levels, starting with the regulation of channel subunit mRNA splicing and channel subunit trafficking (Lipscombe et al., 2013). Much has also been learned about the targeting and cellular distribution of Ca_V_ channels (Herlitze et al., 2003), as well as G-Protein-mediated inhibition of presynaptic Ca_V_ channels (Tedford and Zamponi, 2006; Currie, 2010; Zamponi and Currie, 2013). Across metazoans as diverse as nematodes and humans, cytoplasmic, calcium-binding regulatory messengers such as calmodulin integrate cytoplasmic calcium entry with activation of downstream targets, such as calcium/calmodulin-dependent protein kinases (e.g., CaMK, CaMKII) (Liu et al., 2007)—or even inhibition or facilitation of Ca_V_ channels themselves (Halling et al., 2006; Dunlap, 2007; Catterall and Few, 2008; Minor and Findeisen, 2010; Christel and Lee, 2012). Mitochondria and the endoplasmic reticulum also play conserved modulatory roles at many model synapses, acting as calcium buffers and intracellular sources of calcium (Verkhratsky and Petersen, 1998; Collin et al., 2005; Liu et al., 2005; Williams et al., 2013). Additionally, genetic mutations and toxins that impair Ca_V_ channel function cause numerous cellular defects, including impairment of neurotransmission and alterations of forms of neuroplasticity (Catterall and Few, 2008; Norton and McDonough, 2008). These topics have been studied and reviewed thoroughly elsewhere (including a comprehensive book, Zamponi, 2005).

**Figure 1:**
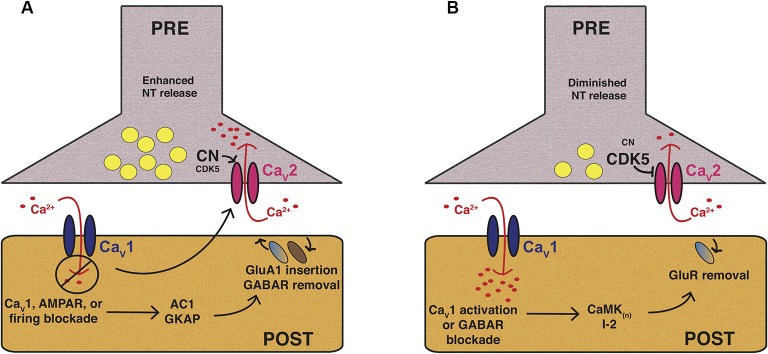
**Mammalian Ca_V_1 and Ca_V_2 channels play central roles in forms of homeostatic plasticity.** These two cartoons attempt to synthesize knowledge of mammalian pre- and postsynaptic homeostatic plasticity mechanisms involving Ca_V_1 and Ca_V_2 calcium channels in preparations like cultured hippocampal neurons. The cartoons are not intended to depict a single synaptic preparation or universally conserved mechanism, though some molecular responses may be widely conserved. **(A)** Homeostatic potentiation of synapse function. Inhibition of synaptic activity or postsynaptic Ca_V_1 calcium influx results in multiple changes, including postsynaptic signaling through molecules like adenylate cyclase 1 (AC1) or guanylate kinase-associated protein (GKAP) to drive activating mechanisms, such as glutamate receptor insertion. Trans-synaptic signaling controlled by factors like Target of Rapamycin (TOR) and Brain-Derived Neurotrophic Factor (BDNF) can trigger enhanced presynaptic release probability. From a variety of systems there is evidence for enhanced presynaptic calcium influx through Ca_V_2—which may require diminishment of cyclin-dependent kinase 5 (CDK5) function—as well as an enhanced readily releasable pool of presynaptic vesicles. **(B)** Homeostatic downscaling of synapse function. Synaptic activation (e.g., through GABA receptor blockade) and/or enhanced postsynaptic calcium influx through Ca_V_1 results in the activation of diverse pathways, such as those mediated by calcium/calmodulin-dependent kinases (CaMK), as well as the Protein Phosphatase 1 (PP1) inhibitor, I-2. This can result in removal of excitatory glutamate receptors from the synapse. Presynaptically, there is evidence of diminished calcium influx through Ca_V_2, and thus, diminished evoked presynaptic release.

**Figure 2:**
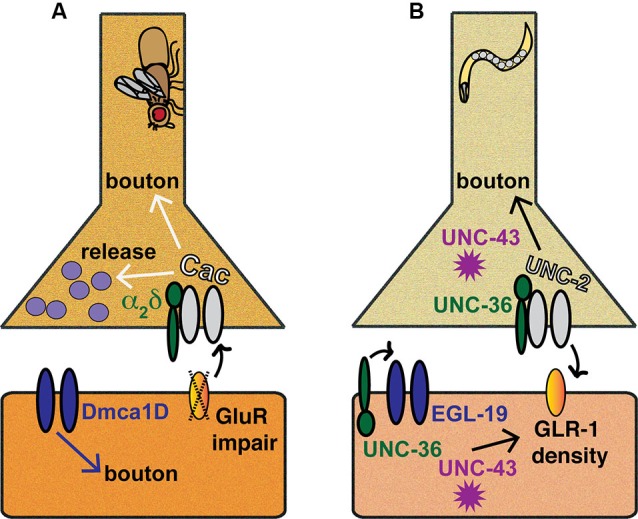
**Invertebrate models of Ca_V_-directed homeostatic plasticity.** Inspection of the *Drosophila melanogaster* NMJ has provided a wealth of information regarding the roles VGCCs and associated molecules play in homeostatic plasticity, as has examination of *C. elegans* preparations, such as the ventral nerve cord (VNC) or the NMJ. **(A)** Drosophila NMJ. Pharmacological or genetic impairment of postsynaptic glutamate receptors triggers a retrograde signaling process that results in enhanced presynaptic Cac/Ca_V_2 function and increased neurotransmitter release. Additionally, Cac/Ca_V_2, α_2_δ, and Dmca1D/Ca_V_1 all affect synaptic bouton development or maturation at the NMJ. **(B)** *C. elegans* synapses. At the *C. elegans* VNC, the coordinated functions of UNC-2/Ca_V_2, EGL-19/Ca_V_1, UNC-36/α_2_δ and UNC-43/CaMKII ensure proper coupling of GLR-1 glutamate receptor density to developmental growth.

With the backdrop of this considerable knowledge, we consider the emerging roles that VGCCs play in HSP. We review electrophysiological, biochemical, and imaging data that have established the important roles Ca_V_ channels play in multiple forms of HSP across diverse experimental systems (Figures 1, 2). We also briefly consider the idea that Ca_V_ channels might link HSP to disorders in which underlying neuronal stability is lost.

## CA_V_2 channels and presynaptic homeostatic synaptic plasticity

Ca_V_2-type calcium channels function in the presynaptic nervous systems of nearly all animals. There are several Ca_V_2 subtypes, classified primarily by sensitivity to different toxins; they include the P/Q- (Ca_V_2.1), N- (Ca_V_2.2), and R-type (Ca_V_2.3) calcium channels (Catterall et al., 2005; Zamponi, 2005). Ca_V_2 channels generally mediate fast neurotransmission, gating presynaptic calcium influx upon cellular depolarization (Catterall et al., 2005; Zamponi, 2005). In turn, calcium-sensing molecules such as Synaptotagmin I trigger rapid evoked neurotransmitter release (Giraudo et al., 2006; Schaub et al., 2006; Tang et al., 2006). Here we review data that demonstrate roles for presynaptic Ca_V_2 channels in HSP at fruit fly and mammalian synapses. In particular, several forms of homeostatic plasticity appear to govern alterations in presynaptic neurotransmitter release by directly targeting the amount of terminal calcium influx through Ca_V_2-type channels.

### Homeostatic plasticity and CA_V_2 at the drosophila neuromuscular synapse

The *Drosophila melanogaster* larval neuromuscular junction (NMJ) is a glutamatergic synapse that exhibits a strong capacity for homeostatic regulation (Frank, 2014; Figure 2A). Several studies have established that the NMJ retains normal levels of postsynaptic evoked excitation, even when it is challenged by chronic or acute perturbations to excitability (Petersen et al., 1997; Davis et al., 1998; DiAntonio et al., 1999; Paradis et al., 2001; Frank et al., 2006). For instance, genetic or pharmacological impairment of muscle glutamate receptors results in decreased sensitivity to single vesicles of glutamate (Petersen et al., 1997; Frank et al., 2006). A homeostatic, retrograde, muscle-to-nerve signaling process helps to offset this decreased sensitivity by inducing a presynaptic increase in vesicle release. This increase has been measured by multiple electrophysiological means (Petersen et al., 1997; Frank et al., 2006). We recently reviewed our understanding of mechanisms underlying homeostatic plasticity at the Drosophila NMJ, including investigations to uncover the unknown retrograde signal(s) and the central roles played by Ca_V_2 channels and presynaptic calcium influx (Frank, 2014). For completeness, some of that same information is reiterated here, but it is updated with newly published data.

#### Cacophony/Ca_V_2

The pore-forming α_1_ subunit of Drosophila Ca_V_2 channels is called Cacophony (Cac; Smith et al., 1996). Loss-of-function mutations in the *cac* gene were originally found in the 1970s from genetic approaches to identify fruit flies with visual defects and defects in mating behavior; indeed, partial loss-of-function *cac* mutant males buzz their wings with a defective, “cacophonous” mating song (Kulkarni and Hall, 1987; Smith et al., 1998). Cac is required throughout much of development. Null *cac* mutant embryos fail to hatch (Kulkarni and Hall, 1987; Kurshan et al., 2009), and *cac* mRNA expression is prominent in the nervous system (Smith et al., 1996). Partial loss-of-function *cac* hypomorphs are viable and fertile, permitting synaptic analyses at larval and adult stages (Smith et al., 1998; Kawasaki et al., 2000; Brooks et al., 2003). Hypomorphic *cac* mutations cause pronounced neurotransmission defects (Kawasaki et al., 2000; Brooks et al., 2003; Frank et al., 2006), and at the third instar larval stage of development, some hypomorphs display mild NMJ growth defects (Rieckhof et al., 2003; Xing et al., 2005). Neuronally expressed Cac-Green Fluorescent Protein (GFP) rescues the lethality of nulls and co-localizes with presynaptic active zone markers (Kawasaki et al., 2004). Transgenic Cac-GFP has proven to be a useful tool in examining active zones and associated proteins (as in Fouquet et al., 2009).

Ca_V_2-type calcium channels also play a critical role in Drosophila NMJ homeostasis. When the NMJs of *cac* hypomorphs are challenged with a pharmacological or genetic impairment of postsynaptic glutamate receptor function, there is no increase in presynaptic neurotransmitter release (Frank et al., 2006). Therefore, not only do *cac* mutants have neurotransmission defects, but the NMJ also fails to maintain muscle excitation at baseline levels (i.e., the already impaired *cac* mutant levels). This phenotype constitutes a block of synaptic homeostasis. One concern about this finding is that *any* mutant with baseline neurotransmission defects might also have defects in the ability to respond to homeostatic challenges. However, it has been demonstrated that several Drosophila mutations cause baseline NMJ neurotransmission defects without impairing homeostatic plasticity (Goold and Davis, 2007; Dickman and Davis, 2009; Younger et al., 2013). Conversely, other mutations or gene knockdowns impair homeostatic plasticity without causing baseline neurotransmission defects (Dickman and Davis, 2009; Marie et al., 2010; Müller et al., 2011; Dickman et al., 2012).

The requirements for Cac/Ca_V_2 during NMJ homeostasis correspond to measureable changes in presynaptic calcium influx. Direct measurement of evoked presynaptic calcium transients reveals that chronic and acute glutamate receptor impairments induce significant (23–30%) increases in presynaptic calcium influx, presumably through Cac/Ca_V_2 channels (Müller and Davis, 2012). A hypomorphic *cac* point mutation, *cac^S^*, blocks this increase (Müller and Davis, 2012). In addition, pharmacological inhibition of the glutamate receptors results in a swift increase in expression of the presynaptic active zone protein Bruchpilot (an ELKS/CAST homolog), as well as an increase in the active zone cytomatrix structure (Weyhersmüller et al., 2011). Together, these results suggest that Ca_V_2 and other active zone proteins are important targets for homeostatic signaling processes. This is logical because small changes in presynaptic calcium influx are well known to correspond to large changes in vesicle release and neurotransmission (Katz and Miledi, 1970).

What might enhance Ca_V_2 function during HSP at the NMJ? Genetic, electrophysiological, and calcium imaging data suggest that presynaptic Ca_V_2 may be targeted by several signaling paradigms that reside on both sides of the synapse. This is not surprising, given that retrograde, muscle-to-nerve signaling governs synaptic homeostasis at the NMJ. What is surprising is how many pieces of this signaling puzzle have emerged in a few short years. Linking those pieces together into a logical framework will be critical as research moves forward.

#### Postsynaptic signaling and Ca_V_2

Following genetic glutamate receptor impairment, a protein-translation-dependent signaling process in the muscle is driven by Drosophila Target of Rapamycin (TOR) and S6 kinase (S6K) to potentiate presynaptic release (Penney et al., 2012). By contrast, two negative postsynaptic signaling factors are the nuclear receptor Importin 13 (Imp13) and the muscle cytoskeletal/matrix support molecule Dystrophin. Impairments of Imp13 or Dystrophin function cause an increase in presynaptic vesicle release (Giagtzoglou et al., 2009; Pilgram et al., 2010, 2011). Further, postsynaptic *imp13* loss of function causes an increase in presynaptic intracellular calcium levels (Giagtzoglou et al., 2009). It is not known if S6K/TOR-, Dystrophin-, and Imp13-mediated signaling events directly interact with one another to regulate homeostatic changes in neurotransmitter release. Given the extensive Drosophila genetic toolkit, this issue is imminently addressable.

#### Presynaptic signaling and Ca_V_2

On the presynaptic side of the synapse, several molecules are hypothesized to directly or indirectly enhance Ca_V_2 function after glutamate receptor impairment. Among these is a signaling system driven by the Drosophila Eph receptor tyrosine kinase, the cytoplasmic guanine exchange factor Ephexin, and Rho-type GTPases (Frank et al., 2009). Loss-of-function mutations in components of this signaling system impair synaptic homeostasis and interact genetically with *cac* mutations (Frank et al., 2009).

The kinesin super family member Khc-73 may work in a similar fashion as Eph/Ephexin. *khc-73* loss-of-function mutations completely impair synaptic homeostasis (Tsurudome et al., 2010), and electron microscopy data demonstrate that Khc-73 contributes to enhancement of active zone components at the NMJ (Tsurudome et al., 2010). A negative regulator of *khc-73* gene expression is the miR-310 micro RNA cluster. Loss of the miR-310 genetic locus phenocopies transgenic overexpression of *khc-73* and shows enhanced presynaptic evoked calcium transients, possibly due to enhanced Ca_V_2 activity (Tsurudome et al., 2010).

Finally, a factor that interacts with calcium channels and assists their localization to the active zone is Drosophila Rab3 Interacting Molecule (RIM; Graf et al., 2012). *rim* loss-of-function mutations block synaptic homeostasis at the NMJ, but not because of a failure to upregulate presynaptic calcium influx. Instead, *rim* mutations occlude important increases in the size of the readily releasable pool (RRP) of presynaptic vesicles (Müller et al., 2012).

#### New data: ENaC and Ca_V_2

A very recent study offers compelling insights into how Ca_V_2 function may be potentiated during synaptic homeostasis. This study demonstrates that presynaptic Epithelial Sodium Channel (ENaC) components encoded by the *pickpocket11* (*ppk11*) and *pickpocket16* (*ppk16*) genes are both required for synaptic homeostasis (Younger et al., 2013). They are also required for the accompanying enhanced presynaptic calcium influx (Younger et al., 2013). These results are drawn not only from genetic mutant data, but also from application of the drug benzamil to impair ENaC (Younger et al., 2013). Based on these new data and previously published data about ENaC function as a voltage-insensitive cation channel (Schild, 2010), one compelling model is that PPK11- and PPK16-ENaC channels act to depolarize presynaptic membrane voltage (Younger et al., 2013). As a result, depolarized resting presynaptic voltage may enhance presynaptic Ca_V_2 activity during homeostatic plasticity.

Arclight is a new genetically encoded voltage sensor that shows robust utility in Drosophila to measure membrane voltage in response to action potentials and sub threshold events (Cao et al., 2013). An attractive possibility for future research is to design new tools—similar to Arclight—that could reliably measure alterations in resting membrane voltage. Such tools could be utilized to directly test the voltage/Ca_V_2 hypothesis for ENaC and possibly to hunt for other factors that control neurotransmission and HSP upstream of Ca_V_2 function.

### CA_V_2 and homeostatic synaptic plasticity at mammalian central synapses

There are similarities in Ca_V_2-gated HSP between the Drosophila NMJ (Figure 2A) and mammalian central synapses (Figure 1). Cultured rodent neurons possess a strong ability to maintain a set point of activity through a homeostatic process termed synaptic scaling (Turrigiano, 2012; Chen et al., 2014; Lee et al., 2014; Pribiag and Stellwagen, 2014; Siddoway et al., 2014; Thalhammer and Cingolani, 2014). It is known that the drugs tetrodotoxin (TTX), 6-cyano-7-nitroquinoxaline-2,3-dione (CNQX), and 2,3-dihydroxy-6-nitro-7-sulfamoyl-benzo[f]quinoxaline-2,3-dione (NBQX) abolish neuronal firing and transmission. TTX blocks sodium channels (Narahashi, 2008), while CNQX and NBQX block postsynaptic AMPA glutamate receptors (Honore et al., 1988; Sheardown, 1993). Conversely, drugs like bicuculline or gabazine inhibit γ-aminobutyric acid (GABA) receptor-mediated inhibitory neurotransmission, and thus, increase activity (Curtis et al., 1970; Uchida et al., 1996). A landmark finding was that chronic application (>48 h) of TTX, CNQX, or bicuculline to cultured rodent visual cortical neurons or to spinal cord neurons elicits a scaling up (TTX and CNQX) or scaling down (bicuculline) of spontaneous miniature amplitudes (O’Brien et al., 1998; Turrigiano et al., 1998). Additionally, drug applications change firing rates in the short run, but upon TTX/CNQX washout or prolonged exposure to bicuculline, activity parameters in exposed neurons homeostatically drive firing rates back in the opposite direction (Turrigiano et al., 1998).

Much work has illuminated the postsynaptic events that acc­om­pany scaling—namely, altered postsynaptic neurotransmitter receptor composition and sensitivity to glutamate (O’Brien et al., 1998; Turrigiano et al., 1998; Wierenga et al., 2005; Sutton et al., 2006; Turrigiano, 2012). However, there has also been a related body of work demonstrating that some central synapses can respond to chronic drug applications in multiple ways. Depending upon the particular preparation or experimental condition, the synaptic response may also be governed through presynaptic changes in RRP size and/or transmitter release (Murthy et al., 2001; Burrone et al., 2002; Thiagarajan et al., 2005). These presynaptic changes do not have to be induced via network-wide perturbations. For example, silencing individual neurons with the inwardly rectifying potassium channel Kir2.1 after synapse formation causes a homeostatic increase of synaptic inputs (Burrone et al., 2002). But what exactly are the presynaptic events that control these responses? Recent results are shedding light on these phenomena.

#### Presynaptic calcium indicators

Presynaptic examinations have been illuminating in defining a role for Ca_V_2 channels in HSP. For example, researchers utilizing a calcium reporter directly localized to synaptic vesicles (SyGCaMP2) demonstrated homeostatic changes in presynaptic calcium influx at rodent hippocampal neurons (Zhao et al., 2011). Synaptic boutons pretreated with TTX for 2 days show a marked increase in presynaptic calcium influx after action potential delivery (Zhao et al., 2011). By contrast, gabazine treatment to inhibit inhibitory GABA_A_ receptors results in decreased presynaptic calcium influx (Zhao et al., 2011). These findings are strikingly reminiscent of those at the Drosophila NMJ (Müller and Davis, 2012). They are also consistent with microscopy data from cultured rodent cortical neurons and the aforementioned Drosophila NMJ, demonstrating that challenges to synaptic activity can result in profound homeostatic changes to components of the presynaptic release machinery (Lazarevic et al., 2011; Weyhersmüller et al., 2011).

#### Retrograde signaling: mTORC1, BDNF, and Ca_V_2

Other results examining hippocampal cultures corroborate this notion. NBQX- or CNQX-mediated blockade of AMPA receptors in the absence of TTX induces two main homeostatic responses: (1) incorporation of new, GluA2-lacking AMPA receptors postsynaptically; and (2) a retrograde signaling process that results in increased presynaptic release properties, such as spontaneous miniature frequency (Thiagarajan et al., 2005; Gong et al., 2007). Similar to the Drosophila system, postsynaptic mammalian Target of Rapamycin Complex1 (mTORC1) drives this retrograde signaling process—albeit through release of Brain-Derived Neurotrophic Factor (BDNF), which is not found in Drosophila (Henry et al., 2012). Also resonant with the Drosophila NMJ, coincident application of a cocktail of N- and P/Q-type calcium channel blockers ω-conotoxin GVIA and ω-agatoxin IVA (CTx/ATx cocktail) completely abolishes the enhanced presynaptic activity induced by 3 h of CNQX exposure (Jakawich et al., 2010). This result supports the idea that presynaptic Ca_V_2 function is required for this form of homeostatic plasticity.

#### Cyclin-dependent kinase 5 (CDK5) and calcineurin

Key components of Ca_V_2.2 (N-type) regulation during HSP appear to be the cyclin-dependent kinase CDK5 and the α isoform of the phosphatase Calcineurin A (CNAα). Inhibition of CDK5 function in CA1-CA3 hippocampal cultures with the drug roscovitine results in enhanced action potential-evoked release and access to the resting synaptic vesicle pool for release (Kim and Ryan, 2010). Consistently, biochemical assays show that chronic silencing of synapses with TTX leads to a measurable decrease of CDK5 (Kim and Ryan, 2010). CDK5 exerts its control of synaptic activity by balancing an opposing function of CNAα (Kim and Ryan, 2010, 2013). The ability of either enzyme to exert control over action potential-driven exocytosis is reliant upon N-type calcium channels, as the N-type blocker ω-conotoxin GVIA occludes their effects (Kim and Ryan, 2013).

## CA_V_1 channels and postsynaptic homeostatic synaptic plasticity

In contrast to Ca_V_2 channels, L-type Ca_V_1 channels are localized to both presynaptic and postsynaptic structures in neurons, as well as non-neuronal excitable tissues—such as skeletal and cardiac muscle—where they are required for excitation-contraction coupling (Hell et al., 1993; Lipscombe et al., 2004; Obermair et al., 2004). Calcium entry though L-type channels activates a variety of downstream calcium-sensitive signaling cascades and gene regulation programs (Lipscombe et al., 2004). There are several sub-types of L-type Ca_V_1 channels, classified by the α_1_ subunit incorporated into the channel: Ca_V_1.1, 1.2, 1.3, and 1.4 (mammalian α_1_ subunit genes *CACNA1S*, *CACNA1C*, *CACNA1D*, and *CACNA1F* respectively). The kinetics of Ca_V_1 activation can be fast (Lipscombe et al., 2004; Helton et al., 2005), however, it is generally thought that slow L-type deactivation allows sustained current, and thus, calcium signaling (McCobb and Beam, 1991; Perrier et al., 2002; Helton et al., 2005). Ca_V_1-type calcium channels are sensitive to dihydropyridine antagonists such as nifedipine or nimodipine, or to phenylalkylamines like verapamil. Neuroscientists typically apply these drugs to electrophysiological preparations in order to assess the results of impaired Ca_V_1 function. These drugs have been used to great effect to show that L-type channels are critical for the expression of a variety of forms of neuroplasticity (Murphy et al., 1991; Magee and Johnston, 1997; Hardingham et al., 1998; Weisskopf et al., 1999; Mermelstein et al., 2000; Brosenitsch and Katz, 2001; Dolmetsch et al., 2001; Lei et al., 2003; Pak and Sheng, 2003)—as well as forms of HSP, detailed below.

### Pioneering findings: chronic blockade of hippocampal neuron activity

In the same study that examined NBQX-induced blockade and the resulting incorporation of GluA2-lacking AMPA receptors in hippocampal cultures, it was reported that nifedipine application mimics NBQX blockade (Thiagarajan et al., 2005). This result suggests that loss of calcium influx through Ca_V_1 channels could gate the mechanism of adding GluA1 homomeric (GluA2-lacking) AMPA receptors to the synapse. To test if Ca_V_1 blockade and AMPA receptor blockade activate a shared mechanism, nifedipine and NBQX were applied concurrently. Dual drug application was indistinguishable from single drug application by biochemical and electrophysiological assays, consistent with a model in which AMPA blockade results in a loss of Ca_V_1 activity (Thiagarajan et al., 2005). In turn, loss of Ca_V_1 activity results in a postsynaptic increase in GluA1 homomers as well as an increase in presynaptic release properties (Thiagarajan et al., 2005). But does Ca_V_1-mediated plasticity occur exclusively after AMPA receptor blockade? No. For example, potentiation of postsynaptic miniature excitatory postsynaptic current (mEPSC) amplitudes also occurs when hippocampal neurons are silenced for 24 h with TTX and allowed to recover in TTX-free saline for a short time before recording (Sokolova and Mody, 2008). In this case, however, TTX/TTX washout-induced potentiation is *occluded* by nifedipine (Sokolova and Mody, 2008).

### How Ca_V_1 silencing induces homeostatic synaptic plasticity

What molecular mechanisms underlie silencing-induced metaplasticity downstream of Ca_V_1? Recent research has implicated many classical calcium-sensitive cytoplasmic signaling molecules and processes. Through biochemistry and electrophysiology, one investigation demonstrated that downstream of CNQX-mediated AMPAR blockade (or blockade of Ca_V_1), adenylate cyclase 1 activates nuclear transcription of new GluA1 subunits, which are then trafficked and incorporated as homomers (Gong et al., 2007). Additionally, a recent study demonstrated that in rat hippocampal neurons, the scaffolding molecule guanylate kinase-associated protein (GKAP) plays a key role in both scaling up synaptic activity (after TTX application) and scaling down of activity (after bicuculline) (Shin et al., 2012). It was found that blockade of Calcium/Calmodulin-dependent Kinase II (CaMKII) activity by the drug KN-93 impairs both the recruitment and the removal of GKAP at synapses. Further examination showed that the source of calcium that results in the activation of CaMKII is important. NMDAR blockade by 2-amino-5-phosphonopentanoic acid (APV) stops bicuculline-driven GKAP removal from synapses; by contrast, Ca_V_1 impairment by nimodipine stops TTX-induced GKAP enhancement at synapses (Shin et al., 2012).

Chronic neuronal silencing not only enhances the abundance of excitatory neurotransmitter receptors in a homeostatic fashion, but it can also decrease the abundance of inhibitory receptors at GABAergic synapses—consistent with network stability working through a balance of excitatory and inhibitory connections (Kilman et al., 2002; Swanwick et al., 2006; Saliba et al., 2007). Electrophysiological experiments have revealed a role for Ca_V_1 in this homeostatic process too. For example, in hippocampal cultures, 24 h of nifedipine application reduces GABAergic synaptic transmission, likely due to an increased turnover and a decreased insertion of GABA_A_ receptors (Saliba et al., 2009).

### Ca_V_1 integrates responses to chronic activation

If loss of calcium influx through postsynaptic Ca_V_1 channels mediates homeostatic forms of potentiation (Figure 1A), could increased calcium influx through Ca_V_1 mediate homeostatic forms of depression (Figure 1B)? Yes—one elegant analysis showed that this is likely true and went further to demonstrate that homeostatic signaling events governed by Ca_V_1 occur not just on the level of entire networks, but also on the level of single cells (Goold and Nicoll, 2010). By taking advantage of optogenetics, the authors of this study excited individual Channelrhodopsin 2 (ChR2)-expressing CA1 pyramidal neurons for 24 h. In response to this excitatory stimulation, there is a homeostatic downregulation of both NMDAR and AMPAR-mediated responses postsynaptically. This compensatory, homeostatic depression works via L-type calcium channels, as nifedipine occludes the effect (Goold and Nicoll, 2010).

### Cellular mechanisms after chronic activation

What are the cellular mechanisms that drive homeostatic downscaling after chronic activation? This question has recently been reviewed (Siddoway et al., 2014). Here, we cover data integrating Ca_V_1 with homeostatic downscaling. In the ChR2-expressing CA1 neurons, this process appears to require Calcium/Calmodulin-dependent protein kinase kinase (CaMKK)—the CaMKK inhibitor STO-609 blocks homeostatic depression (Goold and Nicoll, 2010). This process also requires CaM kinase 4 (CaMK4), as a CaMK4 dominant negative construct also occludes depression (Goold and Nicoll, 2010).

Similar manipulations have uncovered additional calcium-responsive integrators of HSP. In an analysis employing cultured mouse cortical neurons, it was found that chronic bicuculline application causes phosphorylation of the protein phosphatase 1 (PP1) inhibitor I-2. In turn, I-2 phosphorylation is required for appropriate AMPAR trafficking and homeostatic downregulation of synaptic function (Siddoway et al., 2013). L-type calcium channels and calmodulin activity are required for I-2 phosphorylation. Addition of nimodipine or the calmodulin antagonist W7 both block increases in I-2 phosphorylation provoked by bicuculline (Siddoway et al., 2013).

Another example crops up in neocortical neurons, where prolonged hyperactivity induced by gabazine induces increases of production of vesicular glutamate transporter (VGLUT2) and neuronal activity-regulated pentraxin (Narp; Doyle et al., 2010). These increases are hypothesized to be homeostatic in nature because they may lead to increased activation of GABAergic inhibitory feedback neurons (Rutherford et al., 1997; Turrigiano and Nelson, 2004). This form of excitation-transcription coupling is dependent upon Ca_V_1 channels—VGLUT2/Narp induction by gabazine is blocked by nifedipine and verapamil application (Doyle et al., 2010). What acts downstream of Ca_V_1? More classical calcium signaling molecules: VGLUT2/Narp induction is blocked by CaMK antagonists KN-62 and KN-93, as well as mitogen-activated protein kinase (MAPK) antagonists PD98059 and U0126 (Doyle et al., 2010).

## Ca_V_ channels and the homeostatic growth and pruning of synapses

If the end goal of homeostatic plasticity is to keep synapses and circuits functioning within normal physiological ranges, then a logical way to accomplish this task is to add or prune synaptic connections and couple development with activity. The relationship between synaptic growth, developmental plasticity, neurotransmission, and HSP is not entirely understood. The coupling of these processes appears to depend upon the particular system or manipulation examined. With Hebbian forms of plasticity like LTP or LTD, one observes a clear correlation between the growth (potentiation) or shrinkage (depression) of dendritic spines in central synapses (Matsuzaki et al., 2004; Zhou et al., 2004; Tada and Sheng, 2006; Lisman et al., 2012). This is not always the case with homeostatic plasticity. Above we have considered several examples of long-lasting homeostatic mechanisms gated by VGCCs. Many of these involve changes to presynaptic release probability or to postsynaptic neurotransmitter receptor composition, yet do not involve gross morphological changes to synaptic architecture.

Nevertheless, it would be wrong to claim that synapse/circuit function is completely divorced from developmental forms of homeostatic plasticity. The visual systems of mammals and fruit flies offer elegant examples in which developmental homeostatic programs compensate for visual or activity depravation (Mrsic-Flogel et al., 2007; Yuan et al., 2011; Whitt et al., 2014). In the realm of VGCCs and downstream calcium signaling, there are data that suggest direct or indirect control synaptic growth processes that could influence homeostasis. We consider a few examples from both invertebrate and vertebrate systems.

### Drosophila neuromuscular junction (NMJ)

Do long-lasting disruptions of neurotransmission cause synapses to assume alternate, homeostatic developmental programs? Conversely, do developmental alterations result in aberrant neurophysiology? At the Drosophila NMJ, much data suggest that developmental changes are not necessarily coupled to alterations of neurotransmission. A recent study examining evolutionarily diverged species of Drosophila showed that wide variations of “wild-type” synaptic growth—including marked differences in bouton number and branching at the NMJ—redound to indistinguishable physiology (Campbell and Ganetzky, 2012, 2013). These data and the data of others suggest that the properties of synapse growth and function can be uncoupled at insect NMJs.

Concerning Ca_V_ channels, two studies reported that partial *cac* loss-of-function mutations affect synaptic growth by causing a mild decrease in the number of synaptic boutons that are formed (Rieckhof et al., 2003; Xing et al., 2005). Another has shown that null mutations in the α_2_δ subunit gene of Ca_V_2 fail to develop NMJ boutons in embryos (Kurshan et al., 2009). Finally, in a study examining synaptic overgrowth caused by potassium channel mutations, it was found that both postsynaptic L-type Ca_V_1 channels (α_1_ subunit encoded by Drosophila *Dmca1D*) and presynaptic Ca_V_2/Cac channels participate in enabling the overgrowth, at the respective stages of bouton budding (DmCa1D) and maturation (Cac) (Lee and Wu, 2010). It is unclear whether the roles of VGCCs during overgrowth are mechanistically the same as during normal developmental growth.

In all, the aggregate data show that the activity of Drosophila Ca_V_ channels can positively influence NMJ maturation and growth (Figure 2A). In the sense that synapse growth is developmentally coincident with larval growth, this could be considered to be a homeostatic function. However, NMJ bouton developmental phenotypes can clearly be uncoupled from neurotransmission phenotypes. This point highlights the importance of examining both development and electrophysiological responses in this model synapse.

### *Caenorhabditis elegans* synapse development

Ca_V_2 and Ca_V_1 channels govern a number of behaviors studied in nematode worms, such as coordination of normal movement and egg laying (Brenner, 1974; Trent et al., 1983; Schafer and Kenyon, 1995; Lee et al., 1997). In *C. elegans*, the Ca_V_2 α_1_ homolog is called UNC-2 (Schafer and Kenyon, 1995), and the Ca_V_1 α_1_ homolog is called EGL-19 (Lee et al., 1997). *C. elegans* is an excellent system to study Ca_V_ functions because there are strong loss-of-function mutations in the genes encoding these pore-forming subunits, as well as mutations in the genes encoding auxiliary subunits, like the α_2_δ homolog, UNC-36 (Figure 2B). Interestingly, UNC-36 has been shown to be important not only for UNC-2/Ca_V_2 localization and function as would be expected for α_2_δ (Saheki and Bargmann, 2009), but it also modulates the activation and conductance of EGL-19/Ca_V_1-mediated calcium currents (Frøkjaer-Jensen et al., 2006; Lainé et al., 2011).

Throughout *C. elegans* larval development, the density of synapses (the number of synapses per unit length) containing GLR-1 glutamate receptors in the ventral nerve cord (VNC) remains at a set point level, even though absolute synapse number increases dramatically over time (Rongo and Kaplan, 1999). The tight coupling between GLR-1 synapse formation and VNC growth is likely homeostatic, and it is reminiscent of the coupling between organism growth and synapse formation seen at the Drosophila and mammalian NMJs. In *C. elegans*, mutations in the *unc-43* gene—which encodes CaMKII—significantly reduce synaptic density of transgenic GLR-1::GFP protein in the VNCs of adult worms (Rongo and Kaplan, 1999). This indicates an uncoupling between growth and synapse development. This result prompted an investigation into the source of calcium upstream of UNC-43/CaMKII; it was found that *unc-2* and *egl-19* mutations also significantly reduce synapse density (Rongo and Kaplan, 1999). However, in addition to synapse density, GLR-1::GFP puncta intensity was examined in a follow-up study—and here it was found that *unc-2* loss results in a compensatory *increase* in glutamate receptor intensity (Grunwald et al., 2004). Taken together with data from other systems, these *C. elegans* data are consistent with an ancient role in synapse development for both P/Q- and L-type channels.

The importance of CaMKII and Ca_V_ function carries over to the NMJs of *C. elegans*. A recent study demonstrated that mutations in *unc-43*, *unc-2*, or *unc-36* all have altered NMJ morphology (Caylor et al., 2013). A close examination of the NMJs during the L4 larval stage revealed that wild-type NMJs add new boutons in a dynamic process, evidenced by enlarged or elongated puncta of a GFP-tagged Synaptobrevin marker. By contrast, in *unc-2* mutants, this process is muted (Figure 2B; Caylor et al., 2013).

### Striatal medium spiny neurons

Striatal medium spiny neurons (MSNs) offer a vertebrate model of homeostatic control of synaptic growth gated by VGCCs. In MSNs, increased striatal dopamine causes an increase in MSN spine density (Kim et al., 2009). By contrast, decreased dopamine levels (as in Parkinson’s Disease) result in a marked pruning of the MSN spine density, but also a decrease of glutamatergic synapses onto D2 dopamine receptor (D2R)-expressing MSNs (Day et al., 2006; Deutch et al., 2007). Elimination of D2R synapses enhances MSN excitability (Shen et al., 2008). Thus the pruning of D2R-containing synapses likely represents a homeostatic response to the lack of dopamine. Follow-up work employing a combination of potassium-induced membrane depolarization and nimodipine blockage of L-type calcium channels demonstrates that MSN synaptic reduction is dependent upon Ca_V_1.2 function. By contrast, the L-type channel agonist Bay K8644 enhances the effects of membrane depolarization (Tian et al., 2010). Downstream of calcium entry through Ca_V_1.2, there is Calcineurin-mediated activation of Mef2 transcription factor activity (Tian et al., 2010). Consistently, Mef2 has been reported to regulate developmental synaptic remodeling by controlling the expression of a variety of target genes (Flavell et al., 2006, 2008; Fiore et al., 2009; Ye et al., 2013).

## Possible links between Ca_V_ channelopathies and homeostatic plasticity

Ion channel disorders (channelopathies) can have debilitating manifestations (Kullmann, 2010; Ryan and Ptacek, 2010). The fact that stable synapse function depends on homeostatic signaling leads to a logical question: do some channelopathies result from impaired homeostatic systems? The answer is not 100% clear for any disorder. However, several channelopathies—such as forms of epilepsy and migraine—display a possible hallmark of impaired HSP: long periods of neuronal stability followed by sudden, episodic attacks. A series of reviews recently surveyed many calcium channelopathies, including those caused by mutations in subunit genes for Ca_V_1-, Ca_V_2-, and Ca_V_3-type channels (Liao and Soong, 2010; Pietrobon, 2010a; Striessnig et al., 2010; Zamponi et al., 2010; Cain and Snutch, 2011). Another recent review examined compelling connections between HSP and neurological disorders (Wondolowski and Dickman, 2013). Knowing what we know about VGCCs and HSP, we may draw speculative links between some VGCC channelopathies and homeostatic plasticity. Here we consider a subset of them, including episodic forms of migraine, ataxia, myasthenia, epilepsy, and paralysis.

### Ca_V_2.1 channelopathies: migraine, ataxia, and myasthenia

Two Ca_V_2.1 channelopathies—familial hemiplegic migraine type 1 (FHM1) and episodic ataxia type 2 (EA2)—result from mutations (gain- and loss-of-function, respectively) in human *CACNA1A*, which encodes the α_1_ subunit of presynaptic Ca_V_2.1-type calcium channels (Ophoff et al., 1996; Pietrobon, 2010a). A third disorder—spinocerebellar ataxia type 6 (SCA6)—results from poly-glutamine (polyQ) expansion in the *CACNA1A* gene product (Zhuchenko et al., 1997). There is some question about whether SCA6 is more properly classified as polyQ expansion disorder rather than a channelopathy. Here we focus on FHM1 and EA2.

Migraine is the most common neurological disorder—about 12% of the population suffers from it, with a high lifetime incidence rate for women (Barrett et al., 2008; Stewart et al., 2008; NINDS/NIH, 2009). FHM1 is a rare subtype; it is an inherited Ca_V_2.1 channelopathy that causes migraine with an accompanying aura (Ophoff et al., 1996; Pietrobon, 2010a,b). While some gain-of-function *CACNA1A* mutations cause only FHM1, others can lead to additional maladies, such as epileptic attacks (Kors et al., 2001; Pietrobon, 2010a,b; van den Maagdenberg et al., 2010). Cortical Spreading Depression (CSD) is associated with aura and FHM1 migraines. CSD is defined as a wave of depolarization of neural cells along the cerebral cortex, followed a prolonged period of inactivity (Charles and Baca, 2013). CSD can be monitored in real time with Blood-Oxygen-Level Dependent functional Magnetic Resonance Imaging (BOLD-fMRI). It is unclear whether CSD is coincident with types of migraine other than FHM. However, the fact that a sudden wave of cortical depolarization is tightly coupled to a form of headache is consistent with the idea that homeostatic mechanisms keep neuronal functions within normal physiological ranges—and more importantly, that these mechanisms could be impaired in FHM1 migraine sufferers. There exist knock-in mouse models of FHM1 (van den Maagdenberg et al., 2004, 2010). These knock-in models have been a wonderful resource to deduce P/Q-channel properties of FHM1-inducing amino acid substitutions and to establish that the substitutions do represent gains of channel function. Animal models may also prove to be valuable in examining whether forms of migraine correlate with an underlying disruption in HSP.

In contrast to FHM1, EA2 is caused by loss-of-function mutations in *CACNA1A* (Ophoff et al., 1996; Pietrobon, 2010a). Most of the known EA2 mutations are dominant missense mutations that affect the trafficking of channel subunits, but some cause *CACNA1A* splicing defects (Pietrobon, 2010a). Like most forms of ataxia, EA2 is marked by sudden attacks of uncoordinated movement. The episodic nature of these attacks again suggests a possible impairment in homeostatic plasticity—speculation made all the more intriguing given that Drosophila *cac* loss-of-function mutations impair homeostatic plasticity at the NMJ (Frank et al., 2006, 2009; Müller and Davis, 2012). Again, several rodent EA2 models exist, including spontaneous loss-of-function *CACNA1A* mutations in *tottering*, *leaner*, *rolling Nagoya*, and *rocker* mice—as well as mice lacking functional P/Q channels altogether (Pietrobon, 2005, 2010a; Miki et al., 2008; Plomp et al., 2009). Homozygous *CACNA1A ^–/–^* loss-of-function mutant mice display severe forms of ataxia. It may be fruitful to probe these mice (or neuronal cultures derived from them) to check if homeostatic plasticity is disrupted.

Forms of myasthenia cause muscle weakness. For example, myasthenia gravis is an autoimmune disorder that directly affects the NMJ. Antibodies formed against acetylcholine receptors impair muscle function. This occurs despite the NMJ’s apparent attempts to correct the problem via homeostatic increases in presynaptic quantal content—an observation from both human myasthenic muscle and rodent models of myasthenia gravis (Cull-Candy et al., 1980; Plomp et al., 1992, 1994, 1995). A separate, rare form of myasthenia is Lambert-Eaton myasthenic syndrome (LEMS; Marion et al., 1984; Pascuzzi, 2002; Mareska and Gutmann, 2004). Like myasthenia gravis, LEMS is an autoimmune disorder, but it is also a channelopathy because autoantibodies are formed against Ca_V_2.1-type calcium channels (Motomura et al., 2000). Mammalian NMJs appear to be endowed with homeostatic coping mechanisms, much like insect NMJs. However, in the case of LEMS, interfering with the NMJ’s ability to generate sufficient presynaptic calcium influx may cause sufficient stress to cause myasthenic symptoms.

### Ca_V_ mutations and epilepsy

The term epilepsy encompasses a range of seizure-associated maladies. Epilepsy is broadly defined by multiple seizure events in a single individual (NINDS/NIH, 2004). It is likely the most frequently invoked neurological disorder that is hypothesized to be triggered or facilitated by defects in neuronal homeostatic signaling. This is logical because epilepsies are marked by an underlying instability of neuronal function.

In addition to ataxia, Ca_V_2.1-deficient “EA2” mice experience absence seizures—i.e., brief events that are marked by an abrupt arrest in activity, followed by a return to normal activity (Noebels and Sidman, 1979; Jun et al., 1999). Mice that have loss-of-function mutations in genes encoding α_2_δ and γ_2_ calcium channel subunits and a reduction in Ca_V_2.1 channel activity also display absence seizures (Letts et al., 1998; Barclay et al., 2001; Letts et al., 2003). In humans with heterozygous EA2-causing mutations, absence seizures are not common, but there are instances in which they are present (Zamponi et al., 2010).

Ca_V_3.1 and Ca_V_3.2 T-type calcium channels contribute to a spectrum of polygenic epilepsies. Mutations in the Ca_V_3.1 α_1_ subunit gene *CACNA1G* have been implicated in idiopathic generalized epilepsy (IGE) susceptibility (Singh et al., 2007). Likewise, mutations in the Ca_V_3.2 α_1_ subunit gene *CACNA1H* have been implicated in childhood absence epilepsy (CAE) susceptibility (Chen et al., 2003; Vitko et al., 2005). Canonically, T-type calcium channels are responsible for low-threshold spiking activity in thalamic neurons (Llinas, 1988). As T-type calcium channels open, there is a significant increase in local intracellular calcium levels (Errington et al., 2010); thus, one can easily hypothesize that proper regulation of T-type currents is critical to neuronal stability. In several rodent models of absence epilepsy, there is a robust increase in T-type calcium currents (Tsakiridou et al., 1995; Talley et al., 2000; Broicher et al., 2008). This is true not only for rodent models in which Ca_V_3.1 currents themselves are high (Ernst et al., 2009), but also for cases in which a loss of P/Q-channel activity results in an increase in T-channel activity (Zhang et al., 2002). The latter example may illustrate a hierarchy of homeostatic signals, in which overall calcium currents are maintained at the expense of neuronal stability. A similar type of model has been proposed for potassium currents and homeostasis at the Drosophila NMJ (Bergquist et al., 2010).

### Ca_V_1.1 and hypokalemic periodic paralysis

Starting around adolescence, individuals who suffer from hypokalemic periodic paralysis (HPP) experience intermittent episodes of muscle weakness and paralysis that last on the order of 3–24 h in length. The attacks often occur upon waking or after high levels of carbohydrate consumption (USNLM/NIH, 2011). The episodic nature of these attacks and the mild challenges that trigger them could suggest defective homeostasis in muscle cells. Familial HPP type 1 is caused by missense mutations in *CACNA1S*, which encodes the α_1_ subunit of Ca_V_1.1, and most of the mutations alter arginine residues in the S4 voltage sensor (Sipos et al., 1995; Morrill and Cannon, 1999). Further experiments exploiting muscle fibers from HPP1 patients have suggested that these arginine amino acid substitutions induce cation gating pore currents and that HPP1 muscle has a higher concentration of sodium (Jurkat-Rott et al., 2009).

## Concluding comments

Understanding how VGCCs help to execute homeostatic forms of neuroplasticity represents a new area of research. It offers a fresh way to consider why VGCCs are regulated in the ways that they are. In healthy neurons, it is logical that calcium channels are co-opted to gate homeostatic signaling processes. Presynaptically, neurotransmitter release is dependent upon calcium influx or local domains of high calcium concentration. Postsynaptically, many signaling processes are dependent upon calcium that enters the cell through receptor complexes and L-type calcium channels. Neurons, synapses, and circuits across metazoan nervous systems are endowed with powerful homeostatic set points of activity, and small adjustments to calcium channel activity parameters could help fine tune synaptic outputs in a variety of ways.

An important frontier for research will be to understand how Ca_V_ channels interact with intracellular signaling mechanisms and with the activities of other ion channels to control homeostatic set points of excitable cells. What signaling processes directly modulate Ca_V_2 during homeostatic modulations of neurotransmitter release? What postsynaptic cascades are activated or suppressed downstream of calcium influx through Ca_V_1? We have excellent clues at this date from diverse experimental systems reviewed here. Concerning HSP, it will be interesting to see which modes of regulation prove to be universal and which ones prove to be synapse- or organism-specific. With that knowledge in hand, it may be possible to better understand and address numerous phenomena, including Ca_V_ channelopathies.

## Conflict of interest statement

The author declares that the research was conducted in the absence of any commercial or financial relationships that could be construed as a potential conflict of interest.
